# Malignant transformation of post‐radiation induced erosive lichen planus to squamous cell carcinoma

**DOI:** 10.1002/ski2.443

**Published:** 2024-08-24

**Authors:** R. Maxwell Regester, Kevin R. Kwan, Jennifer M. Fernandez, Adam Sutton, Megan Arthur

**Affiliations:** ^1^ Department of Dermatology University of Nebraska Medical Center Omaha Nebraska USA

## Abstract

Radiation therapy is commonly used to treat various types of malignancies during or after radiation. Approximately 95% percent of patients develop common skin manifestations including dermatitis, atrophy and fibrosis. Rare manifestations, including non‐melanoma skin cancers, morphea, cutaneous angiosarcoma and bullous pemphigoid, have been reported post‐treatment. The development of lichen planus (LP) from radiation therapy is exceedingly rare, with only 14 previous cases reported. Of these, none were associated with malignant transformation. Malignant transformation from LP is uncommon, with reported cases mainly in oral manifestations of LP at rates of ∼1%–2%. Classic cutaneous manifestations of LP have not been associated with an increased risk of malignancy. We report a unique case of erosive cutaneous LP with malignant transformation in a previously radiated site. Our case highlights a novel cutaneous adverse event to radiation treatment and emphasises the importance of considering erosive LP on the differential when evaluating recalcitrant erosions in a previously radiated area and to monitor closely for transformation to squamous cell carcinoma.

## Graphical abstract

The development of lichen planus (LP) from radiation therapy is exceedingly rare, with only 14 previous cases reported, none of which were associated with malignant transformation. We report a novel instance of erosive cutaneous LP developing at a previously radiated site, which subsequently transformed into squamous cell carcinoma. This case underscores the need to consider erosive LP in the differential diagnosis of persistent erosions in radiated areas and highlights the importance of vigilant monitoring for malignant transformation.

## 1 INTRODUCTION

Radiation therapy is a commonly utilised treatment option for malignancy. Highly proliferative cells are the most sensitive cell types to radiation‐induced damage. The skin is a self‐renewing organ and therefore, sensitive to the actions of ionization radiation. Various skin manifestations have been reported as sequelae in patients receiving radiation therapy for internal and external malignancies. Approximately, 95% percent of patients have endorsed skin manifestations either during treatment or post‐treatment.^1^ Commonly reported manifestations include atrophy, dermatitis and dermal fibrosis.^1^ Atypical post‐treatment sequelae include non‐melanoma skin cancers, morphea, cutaneous angiosarcoma and bullous pemphigoid.^1^

The development of LP from radiation therapy is exceedingly rare. Lichen planus is a chronic condition of unknown aetiology with cutaneous, mucosal, nail and hair involvement. The typical cutaneous manifestations are flat‐topped, violaceous, pruritic papules and plaques. Mucosal involvement classically presents as white reticulated plaques though it may be erosive. Some studies have postulated the condition to be associated with a CD8+ T cell driven process.^2^ The condition has been associated with clinical sequelae of scarring, stenosis, infection and rarely transformation into malignancy. Limited studies have suggested the risk of malignant transformation of LP to be around 1%–2%, primarily with oral erosive disease.^3^ Contrary to mucosal LP, classic cutaneous LP has not been associated with an increased risk of malignant transformation. We report a unique case of erosive LP with malignant transformation in a previously radiated site.

## 2 CASE

**FIGURE 1:**
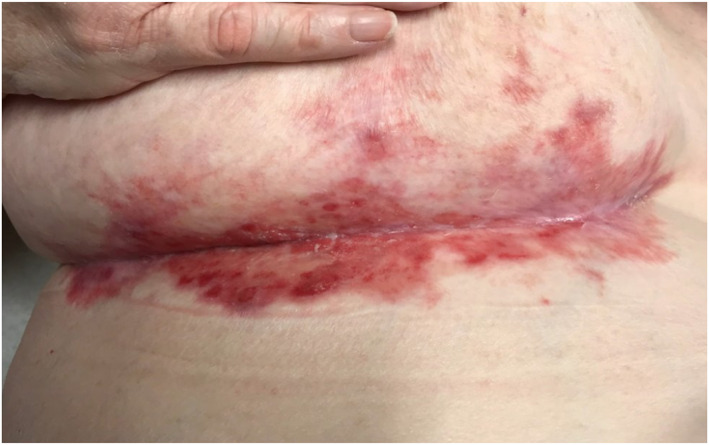
Early findings after the initial diagnosis of erosive LP. LP, lichen planus.

A 71‐year‐old female patient with a history of stage IV chronic kidney disease and right‐sided breast cancer status post lumpectomy and radiation presented to our clinic for worsening erosion under her right breast. The patient reported that the lesion arose shortly following her last dose of radiation. Outside records noted a long history of oral and vulvar LP. The patient was previously evaluated by an outside institution with biopsies both H&E and DIF of the lesion under the right breast showing erosive LP Figure 1 and was trialed on fluconazole, micafungin, hyperbaric oxygen, timolol, intralesional steroids and oral metronidazole without any relief. Temporary relief was achieved with oral prednisone tapers but reoccurred after completion.

**FIGURE 2:**
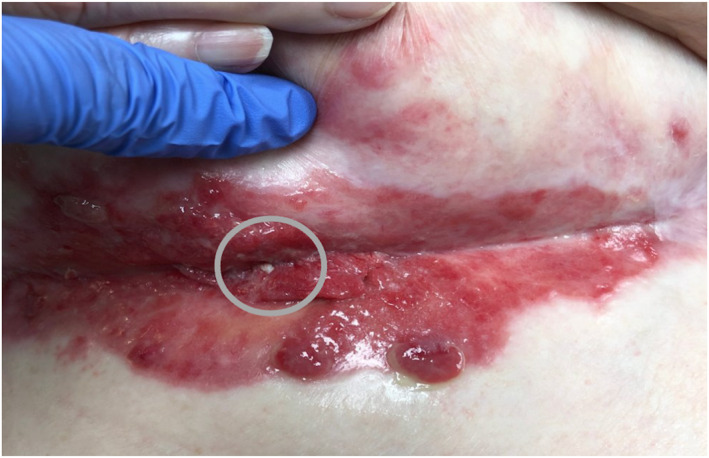
Increasing erythematous friable exophytic plaques within the erosion and new central white hyperkeratotic papule developing.

Physical exam showed erosive and oedematous gingiva consistent with the patient's history of oral LP. No active lesions were seen in the vulvar region. On the right inframammary fold, there was a 14 cm sharply demarcated bright red macerated plaque with ulceration and violaceous lacy changes present at the medial (Figure 2) and lateral aspects.

**FIGURE 3:**
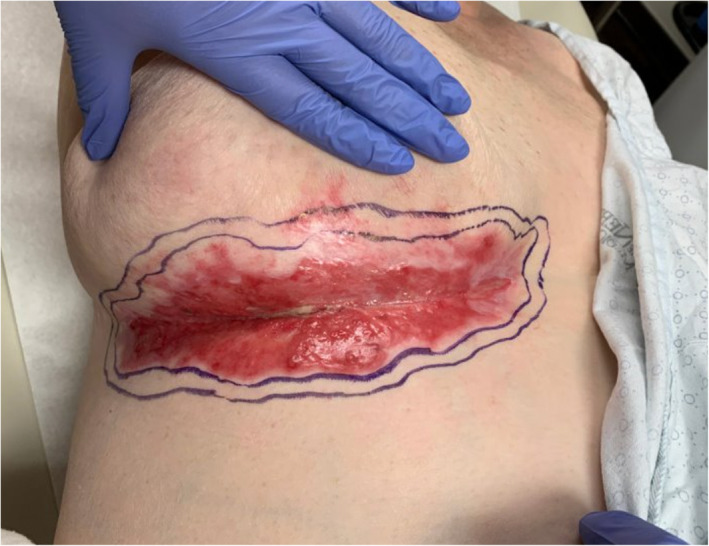
Pre‐excision with comprehensive peripheral and deep margin assessment.

**FIGURE 4:**
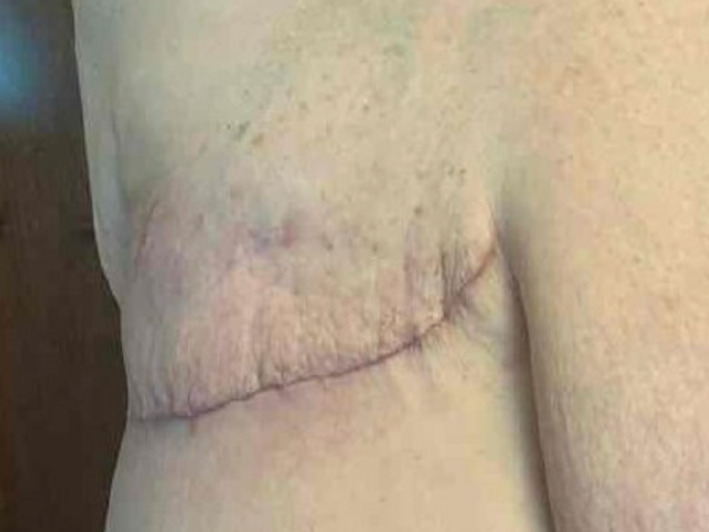
Post‐reconstruction and mastectomy with plastic surgery.

**FIGURE 5:**
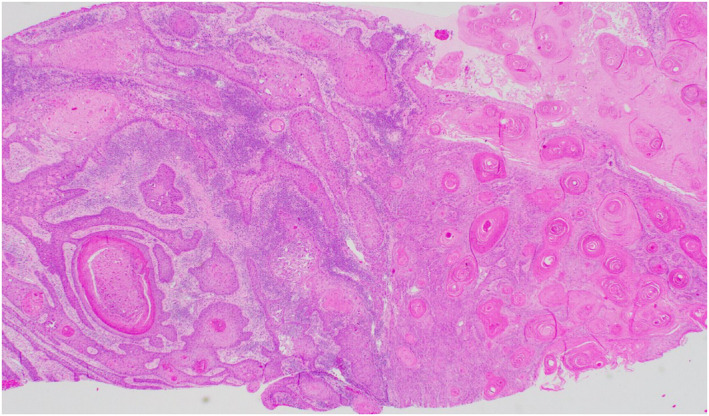
Invasive moderately differentiated SCC present at deep and peripheral margins. SCC, squamous cell carcinoma.

Given the patient's outside records, history and overall clinical appearance, the decision was made to treat for erosive LP. The patient was trialed on metronidazole for 4 months but displayed no improvement and switched to hydroxychloroquine. The treatment plan was further modified to mycophenolate mofetil from hydroxychloroquine after 2 weeks due to the development of a drug rash. Following 3 weeks of continued treatment, the formulation was changed to mycophenolate sodium due to severe gastrointestinal irritation and continued for 7 months. Monthly intralesional Kenalog and topical tacrolimus and clobetasol were utilised in combination with oral treatment through the course of treatment. During medication transition periods, the patient received oral prednisone tapers to aid in temporary relief. At a follow‐up visit, the ulcerated red plaque had enlarged, measuring 25.4 cm × 7.4 cm in overall size, with a worsened friable and hyper‐granulated nodule with a new white hyperkeratotic exophytic papule in the centre (Figure 3). A biopsy was performed on the exophytic lesion, which revealed an invasive moderately differentiated SCC (Figures 4 and 5).

Due to the considerable size of the erosive plaque and the need to accurately assess the extent of SCC before initiating treatment, scouting biopsies were performed. Six scouting biopsies of the plaque under the right breast were performed, which resulted in areas of lichenoid interface dermatitis with no evidence of SCC. An axillary ultrasound was performed to evaluate lymphadenopathy and nodal spread which was negative for reactive lymph nodes nor evidence of lymphadenopathy.

**FIGURE 6:**
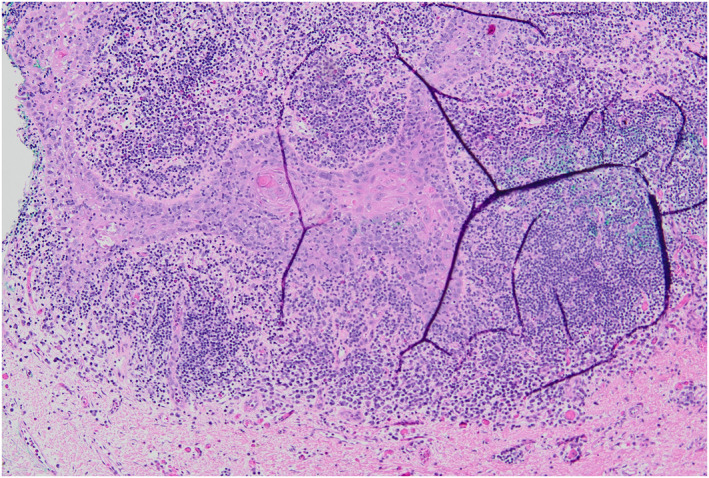
Invasive moderately differentiated SCC zoomed in. SCC, squamous cell carcinoma.

**FIGURE 7:**
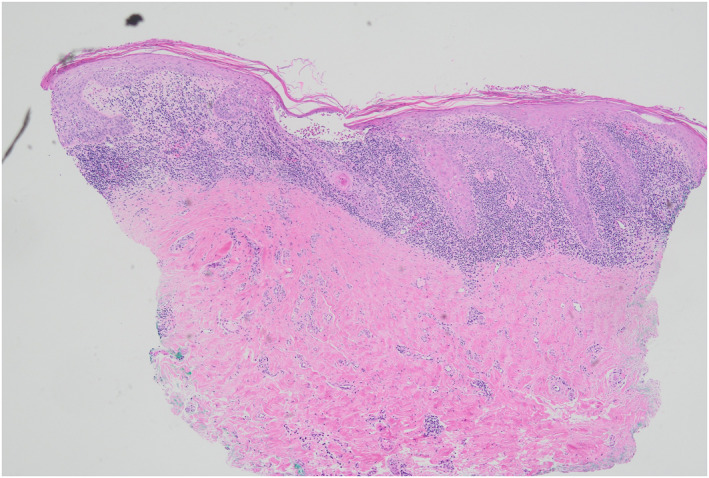
Original histology of lichenoid infiltration.

Given the patient's history of radiation at the site of involvement, overall size of the affected area and moderately differentiated SCC arising within an area of longstanding ulceration, the recommendation was to perform excision with comprehensive peripheral and deep margin assessment and reconstruction with plastic surgery (Figure 6). Complete clearance was achieved with excision surgery with permanent tissue fixation. A mastectomy was performed during the repair procedure with plastic surgery at the patient's request (Figure 7).

## 3 DISCUSSION

The development of any type of LP or lichenoid dermatitis in an area following radiation treatment is extremely rare. An extensive literature search was performed, and a summary of the results of reported cases is listed in Table 1.

**TABLE 1:** Previously reported cases of LP development following radiation therapy.

First author	Age/gender	Underlying malignancy target of radiation therapy	Amount of radiation received (GY)	LP location	LP type	Treatment	Result of LP treatment
Kluger, N	66/F	Lung carcinoma	30	Upper L back, bilateral lower limbs, hands and oral cavity	LP	Betamethasone valerate ointment; 0.1% × 6 weeks–3 months; triamcinolone acetonide mouthwash × 3 months^a^	Complete resolution of skin lesions and great improvement of oral lesions; post‐inflammatory pigmentation
Hopkins, AM	64/F	R breast carcinoma	61	R axilla, lateral breast, IM fold and R shoulder	LP	0.1% triamcinolone ointment 1–2x daily, 3x/week	Well controlled symptoms with return of 2 pruritic papules 10 months s/p treatment
Eichbaum, M	56/F	R breast carcinoma	50.4	R breast, trunk, bilateral forearms and oral cavity	Generalised lichen rubber planus	Initially topical ichthyol, followed by betamethasone propionate; topical tretinoin for oral lesions	5 months s/p radiation; all skin lesions had nearly completely disappeared
Vergilis‐Kalner, IJ	59/F	L breast carcinoma	Not specified	L breast, arm and thigh	LP	0.1% triamcinolone acetonide ointment	Some improvement noted
Pretel, M	44/F	L breast carcinoma	60	L inframammary fold	LP	0.05% clobetasol propionate × 1 month	Resolution of lesions
Komori, T	67/F	Breast carcinoma metastasised to hepatic lymph node and cervical vertebrae	30 to hepatic; 30 to cervical vertebrae	Mid‐back, lower extremities and hands	LP with subsequent development into erosive LP	0.05% difluprednate ointment × 8 weeks to mid‐back; 0.05% clobetasol ointment to extremities	Unresponsive to initial treatment, resolution of lesions with addition of systemic corticosteroids and cessation of nivolumab
Kim, JH	58/M	Thyroid carcinoma involving superior mediastinum	59.4	Neck and anterior chest	LP	0.05% fluocinonide, 0.05% clobetasol propionate at unspecified times over 5 months	Gradual resolution over 5 months; less severe episodic recurrences over the following 6 months
Wang, YN	46/M	Nasopharyngeal carcinoma	66.8	Upper and lower lip and oesophagus	LP	Topical mometasone furoate; oral prednisone 30 mg/day	Resolution of lesions after 5 weeks
Mahajan, R	48/F	Dermatofibrosarcoma protuberans and R lower back	50	R lower back	LP	0.05% clobetasol propionate ointment × 8 weeks	Resolution of lesions after 8 weeks
Sajgane, AA	40/M	Diffuse large B‐cell lymphoma of R knee	45	R knee and L foot	LP with radiotherapy‐induced koebnerization	Not specified	Not specified
Morar, N	67/M	Extramedullary plasmacytoma	45	Scalp, lip, trunk and glans penis	LP	0.05% clobetasol propionate ointment, oral prednisolone 30 mg tapered × 6 weeks	Resolution of lesions after 6 weeks
Shurman,D	68/M	Penile carcinoma	Not specified	Pubic and suprapubic regions, bilateral inguinal and R anteromedial thigh	LP	Topical steroids	“Responded well” to treatment, but patient died secondary to primary cancer
Hadian, Y	66/M	Prostate carcinoma	78	Bilateral glutaeal, lumbosacral and pelvic regions	Hypertrophic LP	0.05% halobetasol ointment daily	Not specified
Rajaintharan, S	87/M	Soft tissue malignancy*	Not specified	R scapular region	LP pemphigoides	Oral prednisolone 30 mg/day for 1 month, tapered over 2 months and cessation of labetalol	Rash subsided over 4 years; recurrence of bullae upon restarting labetalol, which subsided with the same steroid regimen

^a^ GY, grey and LP, lichen planus.

Cases of LP arising in areas of the breast following radiation treatment for breast cancers have been reported,^4^, ^5^, ^6^, ^7^, ^8^, ^9^ but only a single case of the erosive variant of LP in this setting has been reported.^9^ There were no reports of malignant transformation to SCC. Literature reports indicate that lesions generally arise in those who received 50–60 GY of radiation at the affected site.^5^, ^6^ Our case highlights erosive LP with subsequent malignant transformation to SCC in a post‐radiated site, which has not been previously reported in literature.^4^, ^5^, ^10^

Neither the pathophysiology of general LP nor radiation‐induced LP have been elucidated, though studies have implicated an immune‐mediated cause due to an alteration of basal membrane keratinocytes with increased expression of self‐antigens. The self‐antigens trigger an inflammatory response with the activation of CD4+ and CD8+ T cells and cytokine production.^2^ Radiotherapy has been hypothesised to produce lichenoid dermatitis through direct cellular injury triggering the release of self‐antigens from keratinocytes and eliciting an immune response as a result.^4^, ^5^

Classic cutaneous LP has not been shown to have an increased risk for malignant transformation. Erosive LP (oral and genital) and hypertrophic variants have been associated with a low risk for malignant transformation.^11^ Erosive LP creates a tumour‐like microenvironment that contributes to its malignant transformation. Cellular signals and mediators of inflammation (such as interleukin 4 (IL‐4) and IL‐6), implicated in chronic inflammation, play critical roles in increasing the sensitivity of keratinocytes to exogenous mutagens.^2^ Studies have reported the risk of malignancy transformation to range from ∼1% to 2% in patients with erosive LP.^2^, ^3^ Coupled with radiation exposure, which is known to increase the incidence of non‐melanoma skin cancers, the risk of malignant transformation of the erosive LP is likely elevated.

Radiation‐induced LP is a rare consequence of radiation treatment with limited documentation of effective treatment. Most reports have utilised typical treatments for other forms of LP such as corticosteroids, immunosuppressives and phototherapy. No reports have mentioned the management of malignancy arising from radiation‐induced LP. Excision with permanent tissue fixation offers excellent utility as, in this case, the procedure allows for comprehensive margin assessment and accommodates any supplementary immunohistochemistry required, particularly pertinent given the dense lymphocytic inflammation seen in LP.

In summary, we report a unique case of erosive LP in a previously radiated site with sequelae of malignant transformation to SCC. Though extremely rare, it is important to include LP on the differential in a rash developing in an area that previously received radiation therapy. Surveillance of the rash is important as subsequent malignant transformation can occur.

## CONFLICT OF INTEREST STATEMENT

The authors declare that they have no conflicts of interest.

## AUTHOR CONTRIBUTIONS

**R. Maxwell Regester**: Conceptualisation (equal); investigation (equal); methodology (equal); writing – original draft (equal); writing – review & editing (lead). **Kevin R. Kwan**: Conceptualisation (lead); investigation (equal); methodology (equal); writing – original draft (equal); writing – review & editing (equal). **Jennifer M. Fernandez**: Validation (equal); writing – original draft (supporting); writing – review & editing (supporting). **Adam Sutton**: Supervision (equal); validation (equal); writing – original draft (supporting); writing – review & editing (supporting). **Megan Arthur**: Supervision (lead); validation (lead); writing – original draft (supporting); writing – review & editing (supporting).

## ETHICS STATEMENT

The Office of Regulatory Affairs at the University of Nebraska Medical Centre determined that this project does not constitute human subject research as defined at 45CFR46.102. Therefore, it is not subject to federal regulations and does not require IRB review.

## PATIENT CONSENT

Written patient consent for publication was obtained.

## Supporting information

Supporting Information S1

## Data Availability

The data that support the findings of this study are available in the supplementary material of this article.
