# Supplementary material for: Malignant transformation of post‐radiation induced erosive lichen planus to squamous cell carcinoma
Source: Skin Health Dis. 2024 Aug 24;4(6):e443. doi: 10.1002/ski2.443 (PMC11608897; doi:10.1002/ski2.443)
Supplement: Supplementary file 1 — Supporting Information S1 [file SKI2-4-e443-s001.docx]

**Supplementary material**

Supplemental content: Available at <https://data.mendeley.com>
